# ROSIDS23: Network intrusion detection dataset for robot operating system

**DOI:** 10.1016/j.dib.2023.109739

**Published:** 2023-10-31

**Authors:** Elif Değirmenci, Yunus Sabri Kırca, İlker Özçelik, Ahmet Yazıcı

**Affiliations:** a Department of Computer Engineering, Eskisehir Osmangazi University, Eskisehir, 26048, Turkey; b Department of Software Engineering, Eskisehir Osmangazi University, Eskisehir, 26048, Turkey

**Keywords:** ROS, Security, Network traffic, Intrusion detection system, Traffic classification, Network monitoring

## Abstract

The data described herein pertains to the Robotic Systems Security domain. This data in brief presents the attributes of the ROSIDS23 dataset and its collection process in detail. This dataset comprises Robot Operating System (ROS)-based cyber-attacks to address the emerging need in high fidelity data for robotic system security research. The data was gathered from the IFARLab-DIH environment. IFARLab-DIH is a robotic and factory-level laboratory that includes a ROS-based network and is used to conduct studies up to TRL 5 on robotic systems. ROSIDS23 dataset contains benign and various attack traffic collected from the ROS middleware using the tcpdump network protocol analyser. Then the eighty-two traffic features were extracted from the captured pcap files and converted into CSV format using the CICFlowMeter tool. This dataset can serve as a valuable resource for developing and improving security countermeasures in robotic systems and can help the evolution of resilient robotics infrastructure.

Specifications Table

**Table:** 

Subject	Computer Networks and Communications
Specific subject area	Network packets were collected, and traffic features were extracted. Data was obtained from a real robotic system laboratory at stage TRL-5.
Data Format	Raw, Processed
Type of data	Pcap files and CSV files
Data collection	We used a star collection method. Benign and attack traffic was collected via tcpdump in pcap format. The CICFlowMeter tool was employed to extract traffic features and assign labels to the dataset.
Data source location	Institution: Laboratory of center of Intelligent Systems Applications Research, University of Eskişehir Osmangazi City/Town/Region:Eskişehir/Turkiye GPS coordinates for collected samples/data: 39° 44′ 55.61″ N, 30° 28′ 48.84″ E
Data accessibility	Repository name: ROSIDS23 Data identification number: 10.5281/zenodo.10014434 Direct URL to data: https://zenodo.org/records/10014434

## 1 Value of the Data

• The ROSIDS23 dataset contains four security attacks on ROS middleware. Three of these attacks exploit the security weaknesses of ROS. These attacks are targeting availability of ROS middleware nodes.
• This dataset can be utilized for research in fields such as Intrusion Detection Systems (IDS), adversarial attacks analysis, and the Verification and Validation (V&V) processes on industrial systems.
• The data can be used to develop more robust IDS for ROS-based systems. By understanding the patterns of attacks and the vulnerabilities exploited, researchers can devise better prevention techniques and improve ROS's resilience against these security attacks.
• Adversarial attacks can disrupt attack detection systems even with minor distortions. This dataset can assist in designing ROS-based attack detection systems / approaches resilient against adversarial attacks.
• The ROSIDS23 dataset can be used in V&V processes for comprehensively testing and assessing cyber security mechanisms. This can facilitate fine-tuning the defence strategies and effectively addressing potential security vulnerabilities.

## 2 Data Description

The ROSIDS23 dataset was collected from autonomous controlled robotic system network traffic in pcap format. Traffic features were extracted from the collected pcap files using the CICFlowMeter [1]. The dataset includes four security attacks: unauthorized publish, unauthorized subscribe, subscriber flood and DoS. The first three of these attacks are ROS-specific and the final one is general network security attack. The proposed dataset details are given in Table 1. The ROSIDS23 is a multi-class dataset, each row consisting of timestamp, eighty-three features and a label field. The label field can have five different values, namely benign, DoS, unauthorized publish, unauthorized subscribe, and subscriber flood.

**Table 1:** The details of the proposed dataset: ROSIDS23 dataset.

Dataset name:	ROSIDS23
Dataset type:	Multi-class
A total number of features:	83
Number of the classes:	5
List of the class labels:	Benign, DoS, Unauthorized Publish, Unauthorized Subscribe, Subscriber Flood

**Fig. 1:**
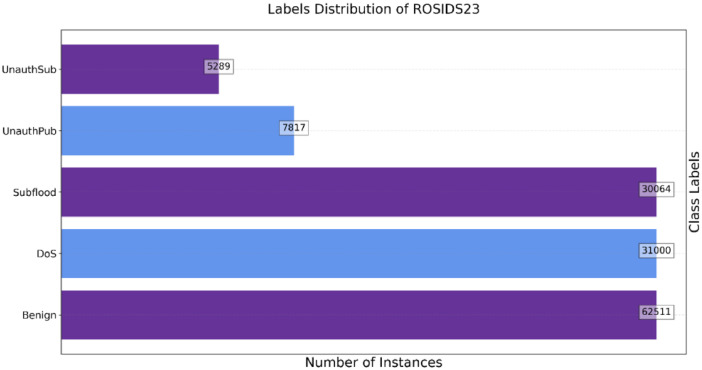
Graphical representation of class distribution of the dataset.

The dataset contains a substantial number of records spanning various categories. Amongst these, the benign records, indicating safe and non-malicious instances, constitute the largest category with 62,511 instances. Denial of Service (DoS) attacks account for 31,000 instances, while Subscriber flood attacks follow with 30,064 instances. Two additional categories highlight specific security concerns: unauthorized subscribe and unauthorized publish. Unauthorized subscribe encompasses instances where unauthorized entities listen or subscribe to a network or data exchange, and it comprises 5289 records. On the other hand, unauthorized publish covers instances where unauthorized entities disseminate or publish information, accounting for 7817 records. Fig. 1 shows the quantity of each type of record in the dataset, providing a clear overview of the dataset's composition.

**Fig. 2:**
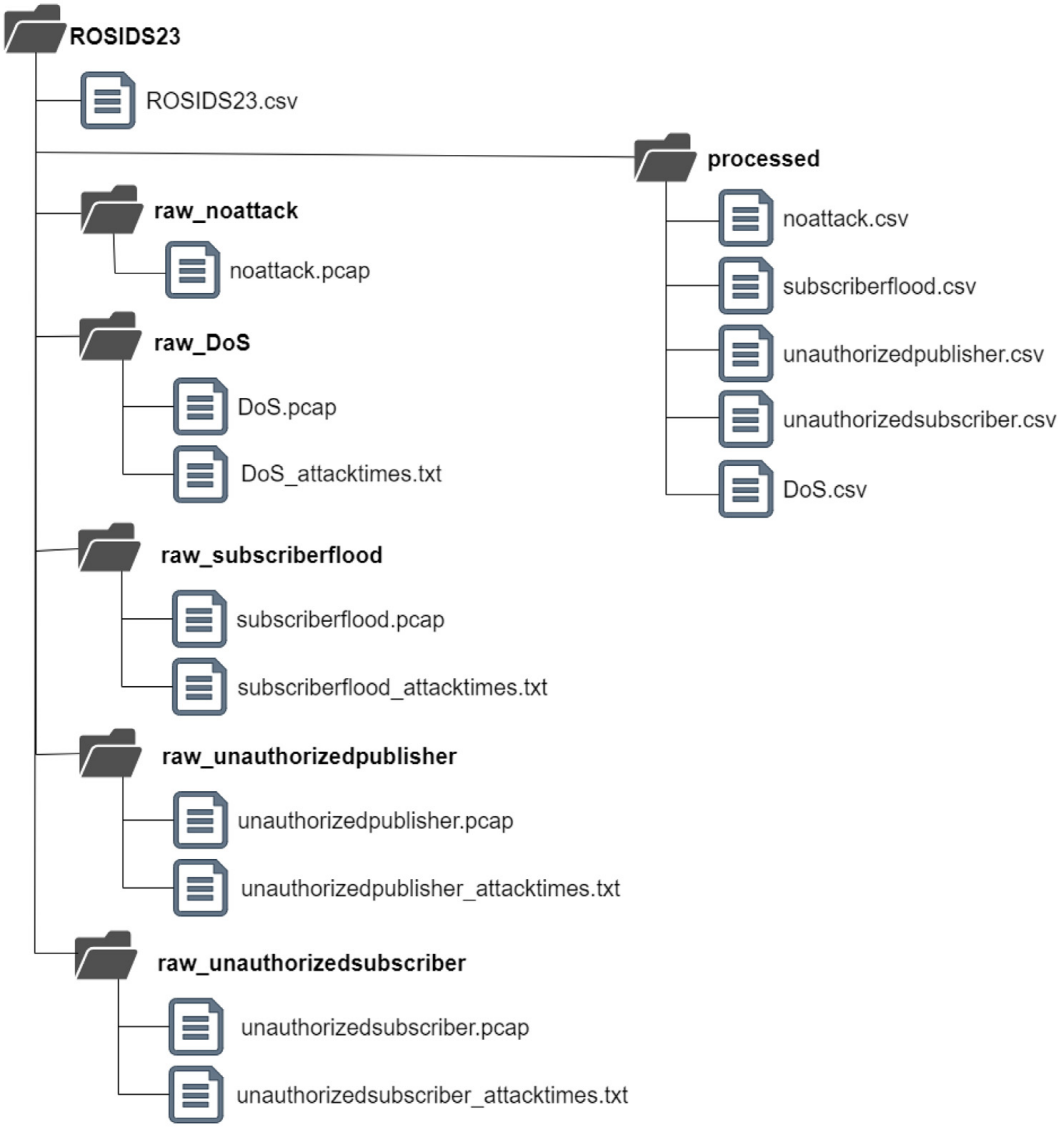
The repository ROSIDS23 dataset file structure.

The dataset consists of raw and processed files. During each network traffic logging session containing an attack, the start time of the each relevant attacks was saved in the '*_attacktimes.txt' file (located under the each related Raw folders).They are published in the Zenodo repository [2]. The repository file structure is given in Fig. 2.

Table 2 provides necessary information about the ROSIDS23 dataset structure including file names, paths, descriptions, and sizes.

**Table 2:** The ROSIDS23 dataset public repository file summary.

File Path	File Name	Description	Size
./	ROSIDS23.csv	This file contains a feature-based multi-class labelled dataset.	72.5 MB
./Raw_ noattack/	noattack.pcap	This file contains benign network traffic captured over a 4 h period with no security attacks. It provides a reference for benign communication between ROS components.	3.2 GB
./Raw_ Subscriber flood/	subscriberflood.pcap	This file captures network traffic over a 60-minute period. A Subscriber attack was evaluated at 1-minute time intervals for a total of 9 min (a ROS-specific security attack).	3.2 GB
./Raw_ unauthorizedsubscriber/	unauthorized subscriber.pcap	This file captures network traffic over a 60-minute period. An Unauthorized Subsriber attack was evaluated at 1-minute time intervals for a total of 9 min (a ROS-specific security attack).	1.9 GB
./Raw_ unauthorizedpublisher/	unauthorized publisher.pcap	This file captures network traffic over a 60-minute period. An Unauthorized Publisher attack was evaluated at 1-minute time intervals for a total of 9 min (a ROS-specific security attack).	1.5 GB
./Raw_DoS/	DoS.pcap	This file captures network traffic over a 60-minute period. A DoS attack was evaluated at 1-minute time intervals for a total of 9 min.	4.3 GB
./Processed/	benign.csv	This file contains only benign traffic features extracted from benign.pcap file. (Not-labelled)	8.7 MB
./Processed/	DoS.csv	This file contains benign and DoS attack traffic features extracted from the DoS.pcap file. (Not-labelled)	318.4 MB
./Processed/	unauthorized subscriber.csv	This file contains benign and unauthorized subscriber attack traffic features extracted from the unauthorizedsubscriber.pcap file. (Not-labelled)	6.3 MB
./Processed/	unauthorized publisher.csv	This file contains benign and unauthorized publisher attack traffic features extracted from unauthorizedpublisher.pcap file. (Not-labelled)	7.1 MB
./Processed/	subscriberflood.csv	This file contains benign and subscriber flood attack traffic features extracted from the subscriberflood.pcap file. (Not-labelled)	236 MB

The proposed dataset contains eighty-four features, with the final column serving as the label (refer to Table 3). The CICFlowMeter tool [1] is used for generating these standard features in the literature [3]. The basic network connection features form the first seven entries: Flow ID, Src IP, Src Port, Dst IP, Dst Port, Protocol, and Timestamp. Features from the 8th to the 20th relate to the network packets. This includes total packets and their length in both forward and backward directions, maximum, minimum, mean, and standard deviation of packet lengths in both directions. From the 21st to the 36th feature, the dataset provides statistical insights about each network flow such as flow rate in bytes and packets per second, mean, standard deviation, maximum, and minimum of inter-arrival times for packets in both directions. The 37th to 57th features are flag counts and header length information, including the number of different TCP flags (PSH, URG, FIN, SYN, RST, ACK, URG, CWE, ECE), lengths of headers in both directions, and the number of packets per second in both directions. The 58th to 66th features provide content-based statistics, including the ratio of download to upload traffic, average packet size, average segment size in both directions, average bytes and packets per bulk, and the average rate of bulk in both directions. Subflow network features are the 67th to 71st features, detailing the number of packets and bytes in a subflow for both directions as well as the number of bytes in the initial window in both directions. The 74th feature captures the number of packets with actual data in the forward direction and the 75th feature provides the minimum segment size observed in the forward direction. The features from 76th to 83rd pertain to the 'active' and 'idle' periods of a flow, including mean, standard deviation, maximum, and minimum time a flow was active or idle. The 84th feature (‘Label’), serves as a classification marker for supervised learning models.

**Table 3:** The ROSIDS23 dataset feature set list.

No	Feature Name	Description	Data Type
1	Flow ID	Unique identifier for the flow of packets.	Integer
2	Src IP	Source IP address from which the packet originated.	Categorical
3	Src Port	Source port of the traffic.	Integer
4	Dst IP	Destination IP address to which the packet is sent.	Categorical
5	Dst Port	Destination port of the traffic.	Integer
6	Protocol	Network protocol used in the packet (TCP/UDP etc.)	Categorical
7	Timestamp	Time at which the packet is transmitted.	DateTime
8	Flow Duration	Duration of the flow in microseconds.	Integer
9	Tot Fwd Pkts	Total packets in the forward direction.	Integer
10	Tot Bwd Pkts	Total packets in the backward direction.	Integer
11	TotLen Fwd Pkts	Total length of packets in the forward direction.	Float
12	TotLen Bwd Pkts	Total length of packets in the backward direction.	Float
13	Fwd Pkt Len Max	Maximum length of packet in the forward direction.	Float
14	Fwd Pkt Len Min	Minimum length of packet in the forward direction.	Float
15	Fwd Pkt Len Mean	Mean length of packets in the forward direction.	Float
16	Fwd Pkt Len Std	Standard deviation of length of packets in the forward direction.	Float
17	Bwd Pkt Len Max	Maximum length of packet in the backward direction.	Float
18	Bwd Pkt Len Min	Minimum length of packet in the backward direction.	Float
19	Bwd Pkt Len Mean	Mean length of packets in the backward direction.	Float
20	Bwd Pkt Len Std	Standard deviation of length of packets in the backward direction.	Float
21	Flow Byts/s	Flow rate in bytes per second.	Float
22	Flow Pkts/s	Flow rate in packets per second.	Float
23	Flow IAT Mean	Mean inter-arrival time between packets.	Float
24	Flow IAT Std	Standard deviation of inter-arrival time between packets.	Float
25	Flow IAT Max	Maximum inter-arrival time between packets.	Float
26	Flow IAT Min	Minimum inter-arrival time between packets.	Float
27	Fwd IAT Tot	Total inter-arrival time of packets in the forward direction.	Float
28	Fwd IAT Mean	Mean inter-arrival time of packets in the forward direction.	Integer
29	Fwd IAT Std	Standard deviation of inter-arrival time of packets in the forward direction.	Integer
30	Fwd IAT Max	Maximum inter-arrival time of packets in the forward direction.	Float
31	Fwd IAT Min	Minimum inter-arrival time of packets in the forward direction.	Float
32	Bwd IAT Tot	Total inter-arrival time of packets in the backward direction.	Float
33	Bwd IAT Mean	Mean inter-arrival time of packets in the backward direction.	Float
34	Bwd IAT Std	Standard deviation of inter-arrival time of packets in the backward direction.	Float
35	Bwd IAT Max	Maximum inter-arrival time of packets in the backward direction.	Float
36	Bwd IAT Min	Minimum inter-arrival time of packets in the backward direction.	Float
37	Fwd PSH Flags	Number of push flags in the forward direction (TCP).	Integer
38	Bwd PSH Flags	Number of push flags in the backward direction (TCP).	Integer
39	Fwd URG Flags	Number of urgent flags in the forward direction (TCP).	Integer
40	Bwd URG Flags	Number of urgent flags in the backward direction (TCP).	Integer
41	Fwd Header Len	Length of the header in the forward direction.	Integer
42	Bwd Header Len	Length of the header in the backward direction.	Integer
43	Fwd Pkts/s	Number of packets in the forward direction per second.	Float
44	Bwd Pkts/s	Number of packets in the backward direction per second.	Float
45	Pkt Len Min	Minimum length of a packet.	Float
46	Pkt Len Max	Maximum length of a packet.	Float
47	Pkt Len Mean	Mean length of a packet.	Float
48	Pkt Len Std	Standard deviation of packet lengths.	Float
49	Pkt Len Var	Variance of packet lengths.	float
50	FIN Flag Cnt	Count of TCP FIN flags.	Integer
51	SYN Flag Cnt	Count of TCP SYN flags.	Integer
52	RST Flag Cnt	Count of TCP RST flags.	Integer
53	PSH Flag Cnt	Count of TCP PSH flags.	Integer
54	ACK Flag Cnt	Count of TCP ACK flags.	Integer
55	URG Flag Cnt	Count of TCP URG flags.	Integer
56	CWE Flag Count	Count of TCP CWE flags.	Integer
57	ECE Flag Cnt	Count of TCP ECE flags.	Integer
58	Down/Up Ratio	Ratio of download/upload traffic.	Float
59	Pkt Size Avg	Average size of the packet.	Float
60	Fwd Seg Size Avg	Average size of the segment in the forward direction.	Float
61	Bwd Seg Size Avg	Average size of the segment in the backward direction.	Float
62	Fwd Byts/b Avg	Average bytes per bulk in the forward direction.	Integer
63	Fwd Pkts/b Avg	Average packets per bulk in the forward direction.	Integer
64	Fwd Blk Rate Avg	Average rate of bulk in the forward direction.	Integer
65	Bwd Byts/b Avg	Average bytes per bulk in the backward direction.	Integer
66	Bwd Pkts/b Avg	Average packets per bulk in the backward direction.	Integer
67	Bwd Blk Rate Avg	Average rate of bulk in the backward direction.	Integer
68	Subflow Fwd Pkts	Number of packets in a subflow in the forward direction.	Integer
69	Subflow Fwd Byts	Number of bytes in a subflow in the forward direction.	Integer
70	Subflow Bwd Pkts	Number of packets in a subflow in the backward direction.	Integer
71	Subflow Bwd Byts	Number of bytes in a subflow in the backward direction.	Integer
72	Init Fwd Win Byts	Number of bytes in the initial window in the forward direction.	Integer
73	Init Bwd Win Byts	Number of bytes in the initial window in the backward direction.	Integer
74	Fwd Act Data Pkts	Number of packets with actual data in the forward direction.	Integer
75	Fwd Seg Size Min	Minimum segment size observed in the forward direction.	Integer
76	Active Mean	Mean time a flow was active before becoming idle.	Integer
77	Active Std	Standard deviation time a flow was active before becoming idle.	Integer
78	Active Max	Maximum time a flow was active before becoming idle.	Integer
79	Active Min	Minimum time a flow was active before becoming idle.	Integer
80	Idle Mean	Mean time a flow was idle.	Integer
81	Idle Std	Standard deviation of time a flow was idle.	Integer
82	Idle Max	Maximum time a flow was idle.	Integer
83	Idle Min	Minimum time a flow was idle.	Integer
84	Label	Label for classification	String

In the literature, widely used public datasets are listed [4], and in this section, we compared them to our proposed dataset (ROSIDS23) in Table 4.

**Table 4:** Public datasets comparisons with proposed ROSIDS23 dataset.

Dataset	Realistic Network	Labelled	Attack Types	Attributes	ROS-based attacks	ROS Environment
KDDCup99	Yes	Yes	DoS, Remote to Local (R2L), User to Root(U2R), Probing	43	No	No
Darpa	Yes	Yes	DoS, Remote to Local (R2L), User to Root(U2R), Probing	41	No	No
NSL-KDD 99	Yes	Yes	Normal, DoS, Remote to Local (R2L), User to Root(U2R), Probing	43	No	No
CICIDS-2017	Yes	Yes	DoS Golden Eye, Bening, DoS hulk, DoS Slow HTTP, DoS Slowloris, DDoS-LOIC HTTP, DDoS-LOIC-UDP, DDoS-HOIC, SSH-Patator, FTP Patator, Brute force, XSS, Botnet, infiltration, SQL injection	84	No	No
CSE-CIC-IDS 2018	Yes	Yes	DoS Golden Eye, Heartbleed, DoS hulk, DoS Slow HTTP, DoS Slowloris, DDoS, SSH-Patator, FP, Patator, Brute force, XSS, Botnet, infiltration, PortScann, SQL injection.	84	No	No
UNSW-NB15	Yes	Yes	Fuzzers, Analysis, Backdoors, DoS, Exploits, Generic, Reconnaissance, Shellcode, Worms	49	No	No
ROSIDS23	Yes	Yes	Subscribing Flood, DoS, Unauthorized Publish, Unauthorized Subscribe	83	Yes	Yes

## 3 Experimental Design, Materials and Methods

**Fig. 3:**
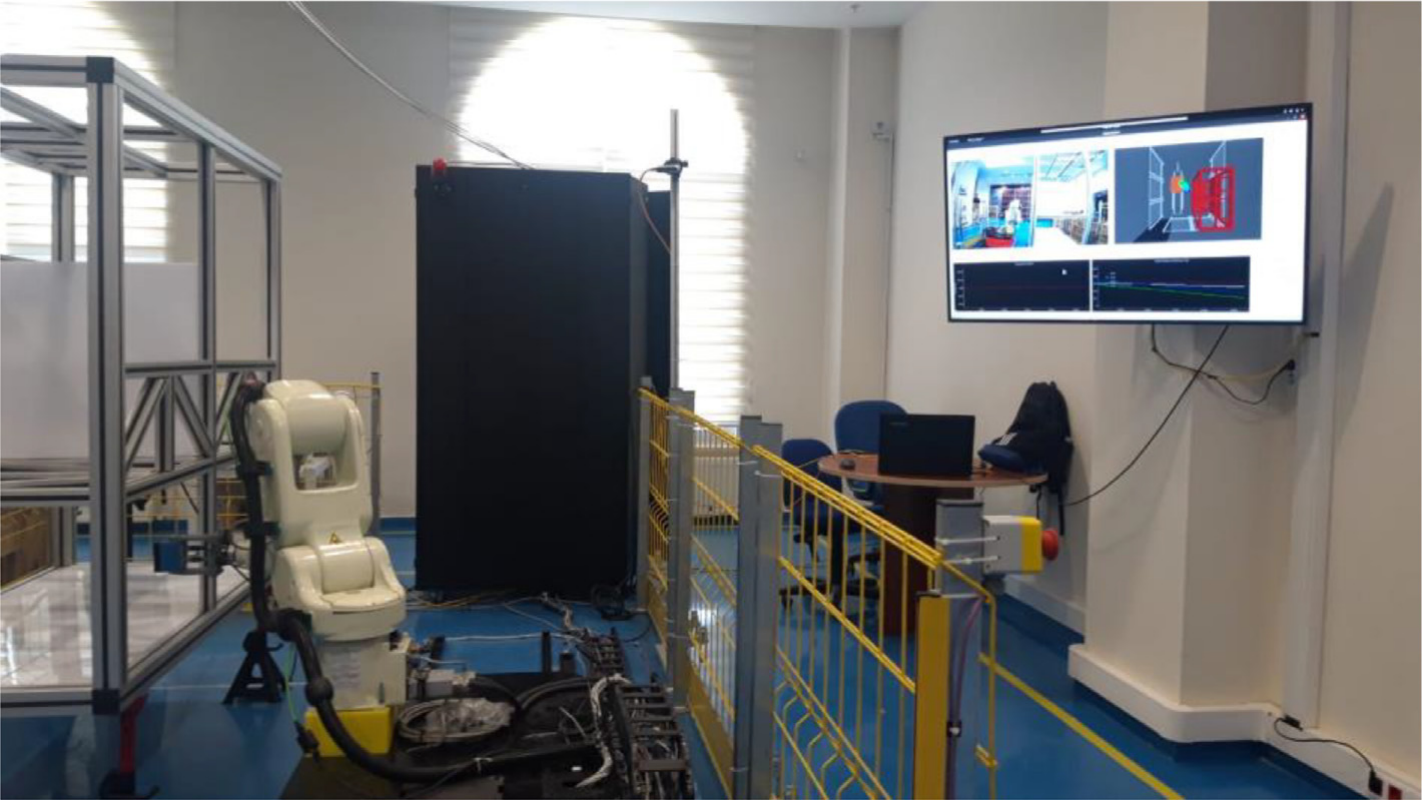
Test-bed Laboratory Environment Setup.

ROSIDS23 is a dataset suitable for intrusion detection studies in ROS-based environments. The dataset was acquired from an experimental system that employs ROS, a widely utilized middleware for robotic Internet of Things (IoT) applications. To collect dataset, a real laboratory environment based on ROS was established at stage TRL5 in the IFARLab-DIH laboratory (Fig. 3). The purpose of this laboratory setup was to test the “Automated Robot Inspection Cell for Quality Control of Automotive Body in White” (ROKOS) [5] within the VALU3S project.

**Fig. 4:**
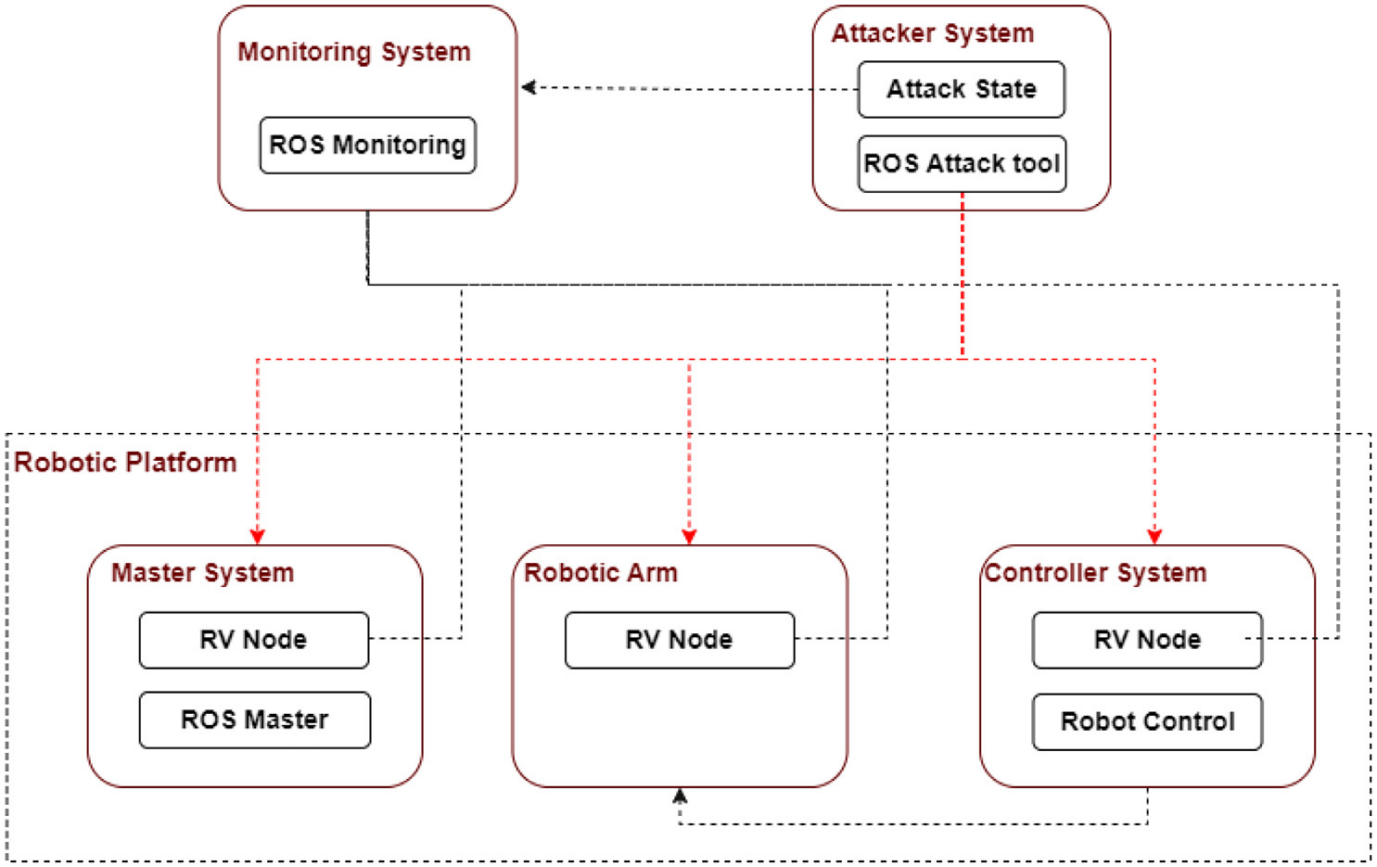
Data collection structure [6].

The laboratory environment hosts the experimental system, which consists of essential ROS components (Master, Controller, and robotic-arm devices), a Logger device for capturing and logging traffic, and an Attacker device for simulating security attacks. This architecture, originally developed for verifying the security of robotic systems in real-time [6], enables comprehensive system monitoring from a centralized location. The same framework, depicted in Fig. 4, was employed for our data collection process.

**Fig. 5:**
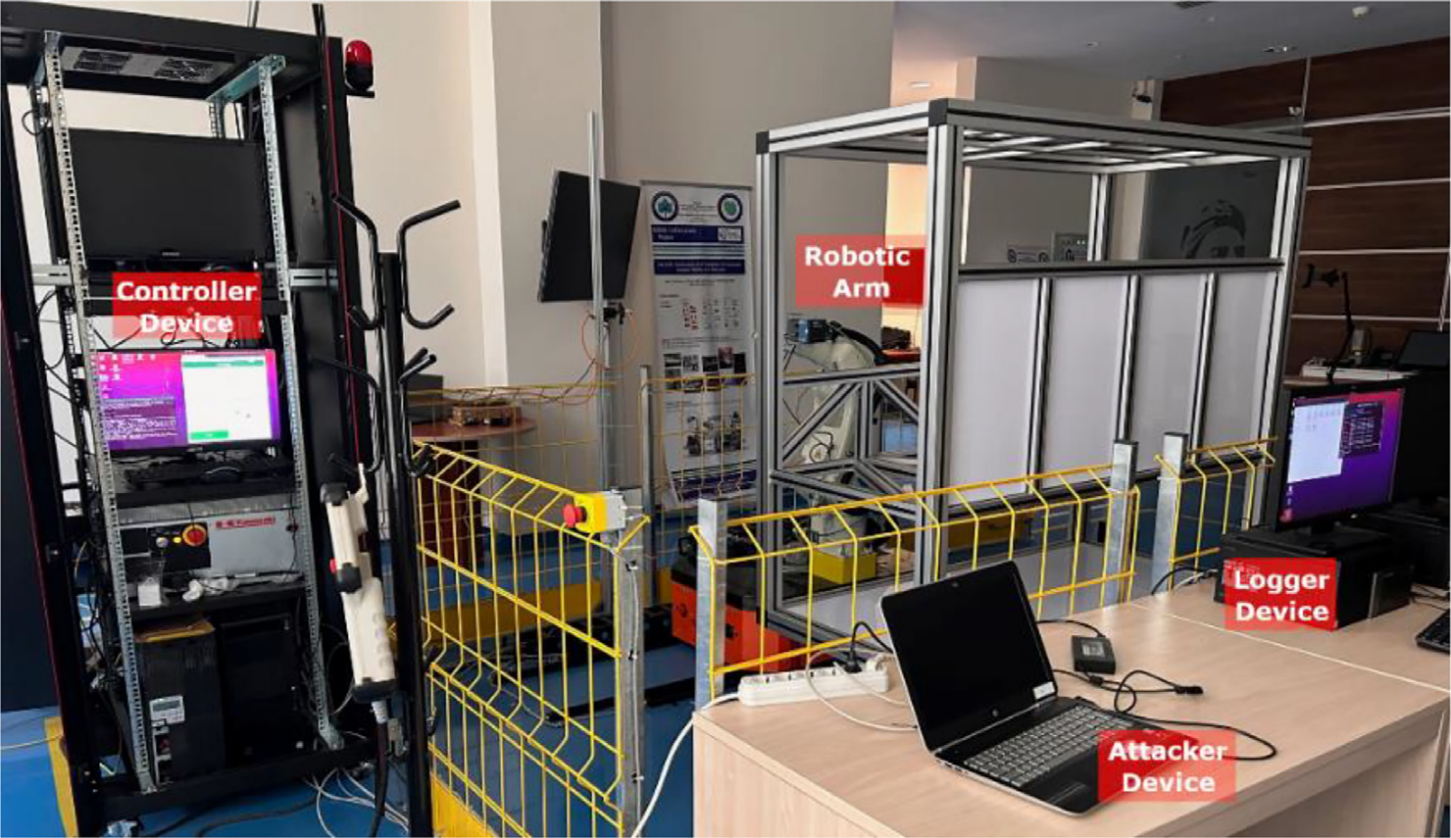
Close view of distributed devices in the laboratory.

The attacks utilized in this dataset, initially published as a preliminary study in [7], demonstrate the impact of DoS attack and other security attacks based on ROS (Robot Operating System) through network layer traffic monitoring. The data collection platform, depicted in Fig. 5, was employed to gather laboratory communication data as benign traffic. The victim network comprised a switch, robotic arm, controller device, and a ROS master. For data collection, the attacker, running Ubuntu 20.04 LTS operating system, was integrated into the data collection platform. Both benign and malicious data were captured using a logger unit.

During the data collection process, network packets were captured using tcpdump, while CICFlowMeter was utilized to extract traffic characteristics from the captured packets. Tcpdump, a packet-based network analyser, collects network packets in real-time [8]. The CICFlowMeter an open-source network traffic flow analyser, helps in extracting features from network flows generated by the ROS middleware. [1].

**Fig. 6:**
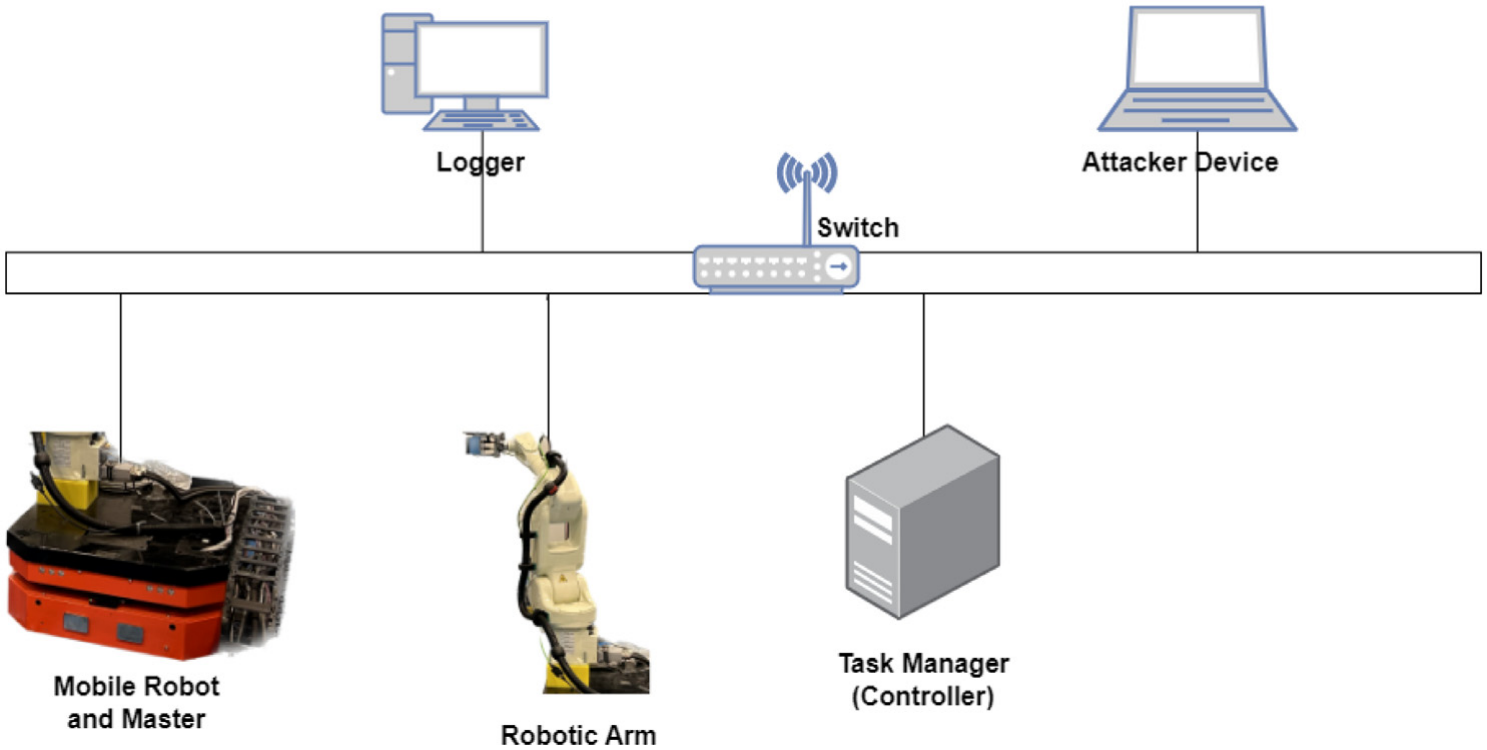
Network traffic star collection design.

Throughout the process of data collection, all packets arriving at the network switch are mirrored to the Logger device. This enables the collection of data at a centralized location, as depicted in Fig. 6. Consequently, even in the absence of communication specifically directed towards the Logger device, it can still monitor the communication occurring amongst the devices within the ROS architecture.

The standard protocol for inter-node communication in ROS commonly involves the use of TCP/UDP protocols. However, the Master device functions primarily using the HTTP protocol, which enables the dissemination of data in XML format. Several operations, such as initiating and deleting nodes, establishing, and terminating inter-node communication, and requesting information about nodes or topics, depend on the HTTP protocol. While ROS can support alternative protocols through customization, this study primarily concentrates on utilizing the default ROS configurations.

The dataset provided encompasses four distinct security breaches that have been extensively discussed in academic research and pose potential risks to authorization in robotic environments. The following descriptions outline these attacks, all of which have been acknowledged and documented in existing literature. Both ROS-specific (ROSAttackTool [9]) and network attack tools were used to carry out attacks. Our evaluation primarily focuses on the Denial of Service (DoS) attack as an assessment of generic threats, while Unauthorized Publish, Unauthorized Subscribe, and Subscriber Flood attacks are examined as ROS-specific threats.

• A Denial of Service (DoS) attack occurs when network resources are utilized to prevent legitimate users from accessing a system. Perpetrators of DoS attacks can execute them by manipulating configuration files of compromised resources, causing physical harm to network components, or excessively consuming system resources [10].
• Unauthorized Publish attack: This attack involves the dissemination of malicious data on ROS. By focusing on specific, crucial areas, an attacker can gain control over the robot, induce its malfunction, or disrupt its intended operation [7]. This attack is performed as if a real ROS communication is taking place using ROS libraries. When the number of attackers increases to high amounts, the nodes responsible for performing robot control can be misled. Additionally, since the attack has a high volume, it can have a DoS-like effect.
• Unauthorized Subscribe attack: In this attack, the attacker gains access to all ROS communications, including the sensor data on the robot. Such data can be utilized for malicious purposes, including the acquisition of personal or confidential company data. The fact that ROS does not include authentication and authorization features makes this attack very easy to carry out. For this attack to occur, it is sufficient to have ROS installed on the attacker device. It does not require an external script. However, in the data set study, in order to make the attack realistic, a method was developed and used to listen to all topics published on ROS at the same time. Furthermore, if there is controller software involved, the extracted data may be manipulated to intentionally cause operational accidents [7].
• Subscriber Flood: This attack constitutes a type of Denial of Service (DoS) attack. In this scenario, the attacker employs numerous fake identities to repeatedly subscribe to a topic published on ROS (Robot Operating System). As the robotic system diligently attempts to handle each incoming request without interruption. Attacker only communicate with ROS Master to prove itself a legit ROS node; on the contrary, he does not use other message packets for any purpose so as not to exhaust his own resources. the excessive demand exhausts the entire bandwidth capacity with ROS Master's and target publisher device's sources [6].

**Fig. 7:**
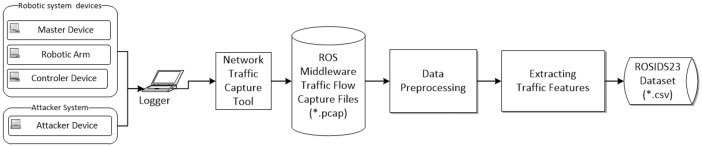
Dataset generation methodology from robotic devices to ROSIDS23 dataset.

The process of dataset creation begins by assessing ROS communication in robotic system devices under normal traffic conditions. Simultaneously, the Attacker system launches its attack. Logger devices capture the ROS traffic by intercepting network packets through the switch mirror and store them using the tcpdump tool. This results in .pcap files containing a complete copy of all ROS network device communication. The workflow for generating the dataset is presented in Fig. 7. Both benign system background traffic and system background traffic with the attack are recorded. Subsequently, the CICFlowMeter tool extracts traffic flow features from the .pcap files, converting more than 80 traffic features into samples stored as .csv files.

The attack scenarios and benign traffic are stored in distinct .pcap files. The benign traffic is recorded as the initial scenario, designated as the benign profile. Each attack scenario is executed individually, and their feature extraction and labelling are performed separately.

## Limitations

The data collection and processing relied heavily on tcpdump and CICFlowMeter tools. Any limitations, biases, or inaccuracies inherent in these tools could potentially affect the dataset.

The dataset only covers four types of attacks: DoS, Unauthorized Publish, Unauthorized Subscribe, and Subscriber Flood. There are numerous other types of attacks that could be performed on ROS-based systems, which were not covered in this dataset.

The study focuses primarily on TCP/UDP and HTTP protocols, which are default in ROS. It may not fully represent systems using other customized or additional protocols.

## Ethics Statements

The present work did not involve the use of human subjects, animal experiments, or data collected from social media platforms*.*

## CRediT authorship contribution statement

**Elif Değirmenci:** Conceptualization, Methodology, Data curation, Writing – original draft. **Yunus Sabri Kırca:** Data curation, Writing – original draft. **İlker Özçelik:** Methodology, Writing – review & editing, Supervision. **Ahmet Yazıcı:** Methodology, Writing – review & editing, Supervision.

## Data Availability

ROSIDS23 (Original data) (Zenodo)
